# Strain-Specific Interactions between the Viral Capsid Proteins VP4, VP7 and VP6 Influence Rescue of Rotavirus Reassortants by Reverse Genetics

**DOI:** 10.3390/ijms24065670

**Published:** 2023-03-16

**Authors:** Roman Valusenko-Mehrkens, Ashish K. Gadicherla, Reimar Johne, Alexander Falkenhagen

**Affiliations:** Department of Biological Safety, German Federal Institute for Risk Assessment, 10589 Berlin, Germany

**Keywords:** rotavirus, reassortment, VP4, VP7, VP6, reverse genetics system, transmission electron microscopy (TEM), replication kinetics, untranslated region (UTR)

## Abstract

*Rotavirus A* (RVA) genome segments can reassort upon co-infection of target cells with two different RVA strains. However, not all reassortants are viable, which limits the ability to generate customized viruses for basic and applied research. To gain insight into the factors that restrict reassortment, we utilized reverse genetics and tested the generation of simian RVA strain SA11 reassortants carrying the human RVA strain Wa capsid proteins VP4, VP7, and VP6 in all possible combinations. VP7-Wa, VP6-Wa, and VP7/VP6-Wa reassortants were effectively rescued, but the VP4-Wa, VP4/VP7-Wa, and VP4/VP6-Wa reassortants were not viable, suggesting a limiting effect of VP4-Wa. However, a VP4/VP7/VP6-Wa triple-reassortant was successfully generated, indicating that the presence of homologous VP7 and VP6 enabled the incorporation of VP4-Wa into the SA11 backbone. The replication kinetics of the triple-reassortant and its parent strain Wa were comparable, while the replication of all other rescued reassortants was similar to SA11. Analysis of the predicted structural protein interfaces identified amino acid residues, which might influence protein interactions. Restoring the natural VP4/VP7/VP6 interactions may therefore improve the rescue of RVA reassortants by reverse genetics, which could be useful for the development of next generation RVA vaccines.

## 1. Introduction

*Rotavirus A* (RVA) is classified as a species in the genus *Rotavirus*, which belongs to the family *Sedoreoviridae* [1]. RVA is able to infect a broad range of hosts, including wild animals, livestock, and humans. Human RVA can cause severe acute gastroenteritis especially in infants and young children. The disease can become life threatening if symptoms, such as severe dehydration, are not treated. Vaccines are available and have reduced the disease burden, but for reasons that are not completely understood yet, their efficacy and effectiveness are reduced in low- and middle-income countries in comparison to high-income countries [2]. Based on data from the years 2017 and 2018, RVA causes over 200,000 deaths per year and is still the leading cause of diarrhea requiring hospitalization in children under five years of age in low- and middle-income countries [3]. 

Rotaviruses are non-enveloped viruses with a triple-layered capsid [4]. The outer capsid layer comprises VP4 and VP7, the intermediate layer VP6, and the inner layer VP2. The protease-sensitive VP4 forms the viral spike and mediates attachment to target cells. Based on the atomic model of an infectious RVA particle [5], each viral spike is comprised of three VP4 molecules. The base of the spike is embedded in the virus capsid and in extensive contact with both VP7 and VP6. The inner capsid layer encloses the viral genome, which comprises eleven different dsRNA segments. Each segment contains one to two open reading frames that are flanked by untranslated regions (UTRs) [6]. Based on the complete nucleotide sequence of all eleven genome segments, a classification system has been established [7]. In this system, the VP7-VP4-VP6-VP1-VP2-VP3-NSP1-NSP2-NSP3-NSP4-NSP5 genotypes are represented by Gx-P[x]-Ix-Rx-Cx-Mx-Ax-Nx-Tx-Ex-Hx, in which x indicates the number of the genotype. Especially the RVA genome segments encoding VP7 and VP4 have a very high genetic diversity. To date, 42 VP7 genotypes (G1-42) and 58 different VP4 genotypes (P[1–58]) have been described [8,9]. Both VP7 and VP4 are involved in viral entry and contain antigenic sites that induce neutralizing antibody responses [10,11,12]. In contrast to the surface-exposed VP7 and VP4, antibodies directed against the intermediate capsid layer protein VP6 are unable to neutralize cell-free mature virus particles. However, it was recently shown that VP6-specific IgG has intracellular neutralization activity and protects mice against rotavirus infection [13].

Zoonotic transmission of RVA between animals and humans has been documented, but viral replication in heterologous hosts is often inefficient [14,15]. Upon superinfection of the same target cell with another RVA strain, an exchange of genome segments is possible, a process referred to as reassortment [16]. Progeny virions generated by reassortment can acquire new phenotypic properties, including improved immune evasion or altered host range [17]. Therefore, reassortment plays a major role in increasing the genetic diversity of RVA. However, little is known about the factors that influence reassortment. Differences in the viral UTRs at the genome segment ends have been shown to limit reassortment between different rotavirus species [18], but within one rotavirus species, at least the outmost genome segment termini are highly conserved. While this could theoretically allow members of RVA to freely exchange genome segments, experiments using reverse genetics (RG) systems suggest that not all genome segments of different RVA strains can readily reassort [19,20,21,22]. Analyses of circulating human RVA strains also indicate that there are preferred genome constellations [23]. In line with this observation, Heiman et al. have shown that human RVA genome constellations are influenced by viral protein interactions and identified genotype specific changes at viral VP2/VP6, VP7/VP6, and VP4/VP6 interfaces [24].

The majority of human RVA strains are difficult to adapt to grow in tissue culture, which has hindered the investigation of factors that influence reassortment. The development of plasmid-based RG systems for RVA [25,26] has enabled easier and faster investigation of the prerequisites for successful reassortment. RG systems are also promising tools for the targeted generation of reassortants for vaccine development. Therefore, multiple studies have examined the reassortment potential of VP4 from human RVA strains in the backbone of the simian RVA strain SA11 using RG systems [20,27,28], leading to successfully generated reassortments with VP4 from tissue-culture-adapted P[8] strains Odelia and CDC-9 as well as from P[4] or P[8] strains of six clinical isolates.

We have previously rescued reassortant SA11 with P[6] VP4 from one clinical isolate that has never been adapted to replicate in tissue culture [29]. However, this was not successful for two other field strains [23]. More surprisingly, we were also unable to rescue reassortant SA11 with P[8] VP4 from the human RVA strain Wa [30], which itself replicates well in cell culture. However, substituting the head region from VP4-Wa, which protrudes from the viral capsid and contains the receptor-binding site, with the corresponding region from VP4-SA11 allowed us to rescue a viable reassortant [31]. This indicates that interaction of the VP4-Wa base with the SA11 VP7 and VP6 capsid proteins is generally possible, but might result in changes in the original VP4-Wa that lead to a highly reduced infectivity. 

In this study, we examined whether interactions between the three capsid proteins VP4, VP7, and VP6 from different strains are important for the generation of viable reassortants. To that end, we tried to rescue reassortant SA11 carrying VP4-Wa, VP7-Wa, and VP6-Wa in all possible combinations. We also compared the replication kinetics of the generated reassortants and parent viruses in tissue culture. The results indicate that interactions between all three proteins can influence the rescue of reassortants.

## 2. Results

### 2.1. Generation of VP7-Wa- and VP6-Wa-Encoding Plasmids

In order to test the hypothesis that strain-specific interactions between the outer and intermediate capsid proteins are important for the generation of viable reassortants, all possible combinations of VP4, VP7, and VP6 should be tested using a well-established plasmid-based RG system [21]. A plasmid encoding VP4-Wa was already available [25]. Plasmids encoding VP6-Wa and VP7-Wa were generated by DNA synthesis and cloning. Whereas the VP6-Wa-encoding plasmid was generated without problems, sequencing analyses showed that the plasmid containing the VP7-Wa genome segment was unstable upon cloning in bacteria. The majority of clones contained deletions, insertions or multiple point mutations in the open reading frame (ORF) of VP7-Wa in independent cloning experiments. The most stable plasmid with minimal sequence changes contained one point mutation in the genome segment (A437G), which resulted in an amino acid substitution in VP7 (D130G). However, predicted structural analysis indicated that the residue was distant from VP4 or VP6 contact sites (Figure S1). Therefore, this construct was used in all subsequent experiments.

### 2.2. Generation of Mono-, Double-, and Triple-Reassortants

**Figure 1:**
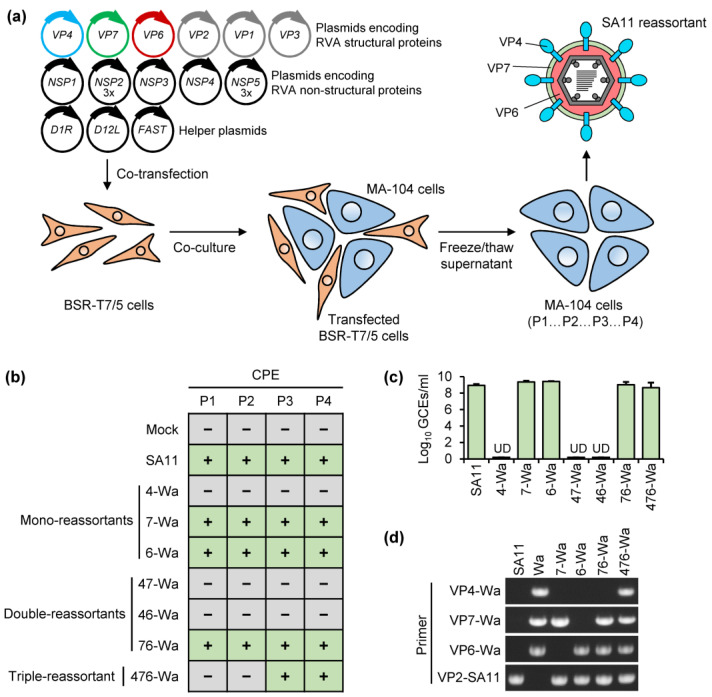
Generation of reassortants carrying VP4, VP7, and VP6 from human RVA strain Wa in the backbone of simian RVA strain SA11. (**a**) Scheme of the RG system used in this study. Eleven plasmids carrying the individual RVA genome segments under transcriptional control of the T7 RNA polymerase promoter as well as three helper plasmids for the constitutive expression of capping enzymes and a small cell fusion protein were co-transfected into BHK-21 cells that express T7 RNA polymerase (BSR-T7/5). Transfected cells were then co-cultured with MA-104 cells and trypsin in the absence of serum. Cell lysates were generated by freezing and thawing and used to infect fresh MA-104 cells. (**b**) Development of a rotavirus-typical cytopathic effect (CPE) upon passaging in MA-104 cells. The result is representative of two independent rescue experiments (**c**) Detection of RVA genomes via qRT-PCR in samples from passage 4 of MA-104 cells. The result is representative of two independent rescue experiments. (**d**) Detection of reassortant-specific genome segments via RT-PCR in samples from passage 4 of MA-104 cells. Abbreviations: 4 = VP4; 7 = VP7; 6 = VP6; CPE = cytopathic effect; P1–P4 = passage 1–4; UD = undetected; GCE = genome copy equivalents.

The experiments to generate mono-, double-, and triple-reassortants of SA11 containing VP4-Wa, VP7-Wa, and VP6-Wa were performed using a plasmid-based RG system, which is schematically shown in Figure 1a. The presence of replication-competent reassortants was determined by passaging in MA-104 cells in all cases. After the first passage in MA-104 cells, a rotavirus-typical cytopathic effect (CPE) was evident for the mono-reassortants with VP7-Wa or VP6-Wa as well as with the VP7/VP6-Wa double-reassortant. For the VP4/VP7/VP6-Wa triple-reassortant, a faint CPE developed during the third passage, which was more clearly visible in the fourth passage. In contrast, no CPE was visible even after the forth passage for the VP4-Wa mono-reassortant, the VP4/VP6-Wa double-reassortant, and the VP4/VP7-Wa double-reassortant. An overview of the CPE observations for all tested reassortants is shown in Figure 1b. After the fourth passage, MA-104 cells including the cell culture supernatant were frozen and thawed. RNA was extracted from clarified supernatants and analyzed by qRT-PCR using RVA NSP3-specific primers and a probe. RVA-RNA was present in all samples from cells with a CPE, while no RVA-RNA was detected in samples from cells without a CPE (Figure 1c). All rescue experiments were repeated one more time with similar results.

**Figure 2:**
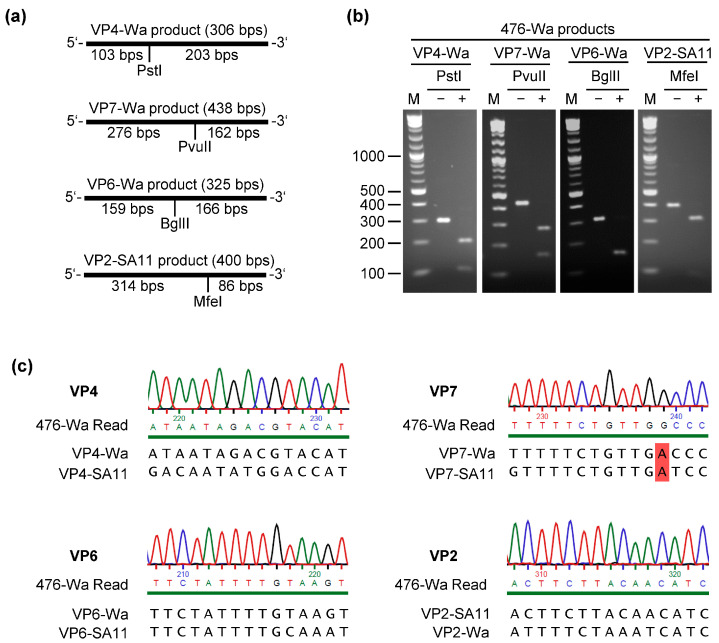
Analyses of RT-PCR products of the VP4/VP7/VP6-Wa triple-reassortant (476-Wa). (**a**) Expected size and position of restriction sites. Bps = base pairs. (**b**) Agarose gel analyses of restriction digests. The numbers indicate the size of the DNA marker fragments (M) in bps. (**c**) Sanger sequencing results. Examples of the chromatograms from the 476-Wa sequencing read are shown. The corresponding Wa and SA11 nucleotide sequences are depicted below each chromatogram. The position at which VP7 from 476-Wa differs from both VP7-Wa and VP7-SA11 is highlighted in red. The green line below the chromatograms indicates that the probability for a wrong base call was equal to or less than 1 in 1000.

**Figure 3:**
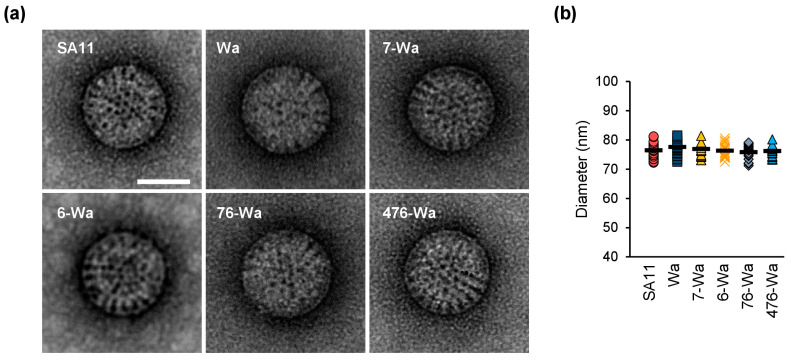
Transmission electron microscopy (TEM) analyses of the generated reassortants and their parent viruses. (**a**) TEM images of negatively stained samples. Scale bar = 50 nm. (**b**) Measurement of triple-layered particle size based on TEM images of negatively stained samples. Abbreviations: 4 = VP4; 7 = VP7; 6 = VP6.

To verify that the intended reassortants were rescued, RNA from the parent RVA strains and from the reassortants was analyzed by RT-PCR using primer pairs specific for VP4-Wa, VP7-Wa, VP6-Wa, or VP2-SA11. Analysis of the resulting PCR products by agarose gel electrophoresis indicated that the expected genome fragments were present for each reassortant (Figure 1d). The PCR products of the VP4/VP7/VP6-Wa triple-reassortant were subsequently purified from the gel and digested with restriction enzymes. The size of the amplicons and relative position of the restriction sites are shown in Figure 2a. After the separation of the undigested and digested samples by agarose gel electrophoresis, the expected fragment pattern was evident for each genome segment (Figure 2b). Additionally, the purified PCR products of the triple-reassortant were analyzed by Sanger sequencing. Examples of the sequencing chromatograms are shown for each fragment in Figure 3c. The sequencing results were identical to the expected sequence, including the A437G mutation in the genome segment encoding VP7-Wa.

### 2.3. Transmission Electron Microscopy (TEM) Analyses

Infectious rotavirus particles have a distinct structure with three layers, referred to as triple-layered particles (TLPs). Non-infectious double-layered particles (DLPs), which lack VP4 and VP7, are smaller in size and have a particle surface that appears rough [32,33]. To examine virus particle morphology, the generated reassortants and their parent viruses were analyzed by TEM. In all samples analyzed, the majority of virions were TLPs without any visible distortion of structure (Figure 3a). The size of 20 random particles per sample was measured (Figure 3b). The average diameter excluding VP4 was 76.4 ± 3 nm, 77.6 ± 2 nm, 76.9 ± 3 nm, 76.3 ± 2 nm, 75.6 ± 2 nm, and 76.2 ± 2 nm for SA11, Wa, the VP7-Wa mono-reassortant, the VP6-Wa mono-reassortant, the VP7/VP6-Wa double-reassortant, and the VP4/VP7/VP6-Wa triple-reassortant, respectively.

### 2.4. Replication Kinetics

**Figure 4:**
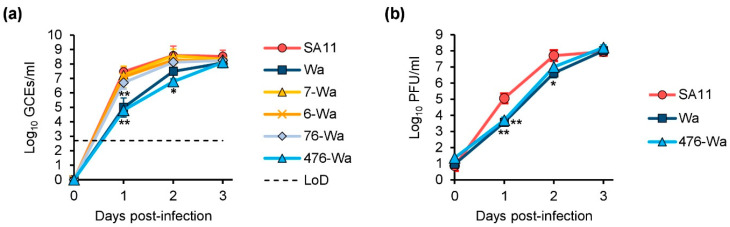
Replication kinetics in MA-104 cells. (**a**) Cells were infected with 2 × 10^4^ GCEs and the number of GCEs/mL was determined by qRT-PCR at the indicated time points post-infection. Results are representative of three independent experiments. ** *p* < 0.01 for Wa or 476-Wa versus SA11. * *p* < 0.05 for 476-Wa versus SA11. (**b**) Cells were infected with a multiplicity of infection (MOI) of 0.003 and the number of PFUs/mL was determined by plaque assay. Results are representative of three independent experiments. ** *p* < 0.01 for Wa or 476-Wa versus SA11. * *p* < 0.05 for Wa versus SA11. Abbreviations: LoD = limit of detection; 4 = VP4; 7 = VP7; 6 = VP6.

In order to investigate the growth kinetics of the generated viruses, we infected MA-104 cells with the reassortants or their parent viruses using equal genome copy equivalents (GCEs). Tissue culture supernatants were collected at multiple time points post-infection and the number of GCEs/mL was determined by qRT-PCR (Figure 4a). The replication kinetics of SA11 and the VP7-Wa and VP6-Wa mono-reassortants, as well as the VP7/VP6-Wa double-reassortant, were highly similar. No statistically significant titer differences between these samples were detected at any time point. In contrast, Wa and the VP4/VP7/VP6-Wa triple-reassortant replicated remarkably slower. At 1 day post-infection (dpi), their titer was over 2.0 log_10_ lower in comparison to SA11 (*p* < 0.01 for Wa versus SA11 and *p* < 0.001 for the triple-reassortant versus SA11). At 2 dpi, the titer differences were less apparent, but still statistically significant for the triple-reassortant (*p* < 0.05). At the endpoint of the experiment, the mean number of GCEs/mL was similar for all examined viruses. During the entire course of the experiment, no statistically significant differences in the titers between Wa and the triple-reassortant were observed.

To further examine the growth kinetics of the triple-reassortant, plaque assays were performed. MA-104 cells were infected with SA11, Wa, or the triple-reassortant using an equal number of plaque-forming units (PFUs). Tissue culture supernatants were again collected at multiple time points post-infection and the number of PFUs/mL for each sample was determined by plaque assay (Figure 4b). Similarly to the previous experiment, Wa and the triple-reassortant replicated slower than SA11. The highest difference was again observed at 1 dpi, at which the PFU/mL for Wa and the triple-reassortant were approximately 1.5 log_10_/mL lower in comparison to SA11 (*p* < 0.01). The titers were still lower at 2 dpi, but the difference did not reach statistical significance for the triple-reassortant. After 3 dpi, all strains grew to a similar infectious titer of 8.0 log_10_ PFU/mL. At any time point, PFUs/mL were not significantly different between Wa and the triple-reassortant. However, it is of note that clear plaques developed 1 day sooner for the triple-reassortant in comparison to Wa, suggesting that there are some differences that are not reflected by the infectious titer.

### 2.5. Untranslated Region (UTR) Sequence Analyses 

Rotavirus UTRs contain sequence and structural elements that play regulatory roles and are involved in genome packaging. The VP4, VP7, and VP6 ORFs are flanked by UTRs of varying length (Figure S2). The outmost termini of the Wa and SA11 genome segments encoding VP4, VP7, or VP6 are highly conserved, but a comparison of the entire UTR sequences revealed some sequence differences between Wa and SA11 (Figure S2), of which the majority were located in the 3′UTRs. While the 5′UTRs of VP4 were identical, the 5′UTRs of VP7 and VP6 differed at 3/49 and 2/23 nucleotides, respectively. The 3′UTRs of VP4, VP7, and VP6 from Wa and SA11 were different at 6/22, 6/37, and 41/139 nucleotide positions, respectively. The significance of these differences is unclear, but they did not seem to negatively affect the replication of the VP4/VP7/VP6-Wa triple-reassortant in comparison to Wa, or that of the VP7-Wa, VP6-Wa, or VP7/VP6-Wa reassortants in comparison to SA11.

### 2.6. Protein Interface Analyses

**Figure 5:**
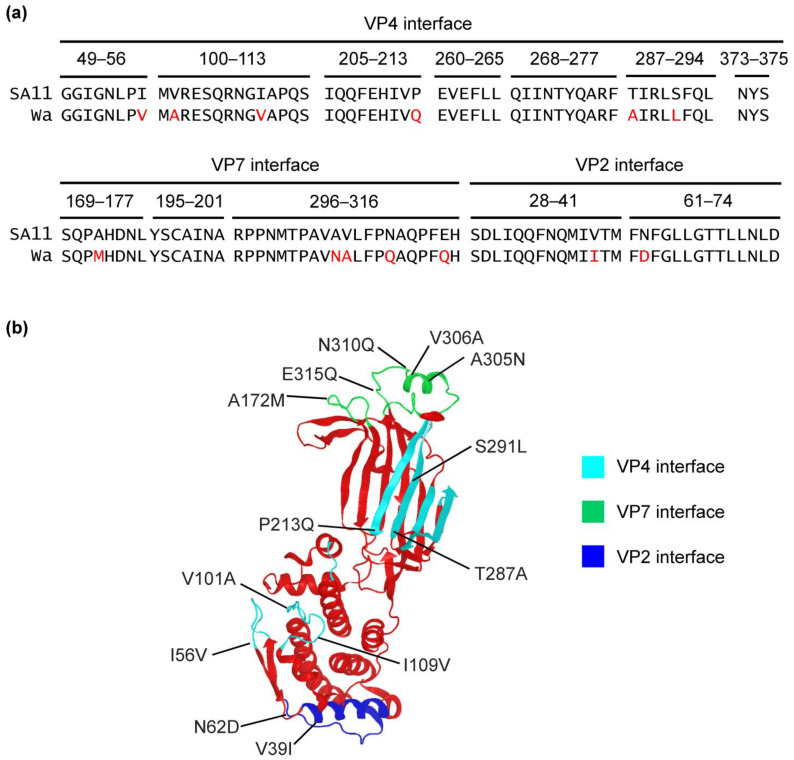
VP4, VP7, and VP2 protein interfaces of VP6. (**a**) Alignment of the amino acid residues located in the interaction sites of VP6 with VP4, VP7, and VP2. The numbers refer to the positions of the amino acid residues that are interfacing with the indicated viral protein. Differences between SA11 and Wa are highlighted in red. (**b**) Location of the identified differences between SA11 and Wa in the atomic structure of VP6 from rhesus rotavirus [5].

As reassortants containing Wa-VP4 could not be rescued without the simultaneous presence of VP7-Wa and VP6-Wa, interactions between all three proteins seem to be important. Regions of VP6 that are in contact with VP4, VP7, and VP2 have previously been analyzed in detail [34]. While VP6 is highly conserved among RVA, amino acid changes in these regions have been shown to influence rotavirus genome segment constellations [24]. VP6-SA11 and VP6-Wa are identical at 365/397 (92%) amino acid residues. However, an amino acid sequence alignment of the VP6 regions that are in contact with VP4, VP7, and VP2 showed that multiple amino acid residues were different among SA11 and Wa (Figure 5a). This includes six amino acid residues at the VP4 interface, four at the VP7 interface, and two at the VP2 interface. Some of them represent changes in polarity (N62D, E315Q), hydrophobicity (A305N, S291L, T287A), or rigidity (P213Q), which could alter VP6 protein interfaces. Figure 5b shows the location of the VP4, VP7, and VP2 contact sites of VP6 and the identified differences.

VP7-SA11 and VP7-Wa are identical at 268/326 (82%) amino acid residues (Figure S3). Amino acid residue differences are located both at the outer surface of VP7 (Figure S4a) and the inner surface that is in contact with VP6 (Figure S4b). Out of these, four differences (T178S, D179G, A181S, and A278N) are in close proximity to the VP6 interface and seven differences (N96G, S97D, L208Q, D211N, A212V, T213D, and T214S) map to two loops on the VP7 outer surface that are in contact with VP4 (Figure S4c). Additionally, four differences (I55L, I65A, A66V, and A68T) are present in the N-terminal VP7 arm [35], which is not only locking VP7 onto VP6, but is also in close proximity to VP4 (Figure S4c).

**Figure 6:**
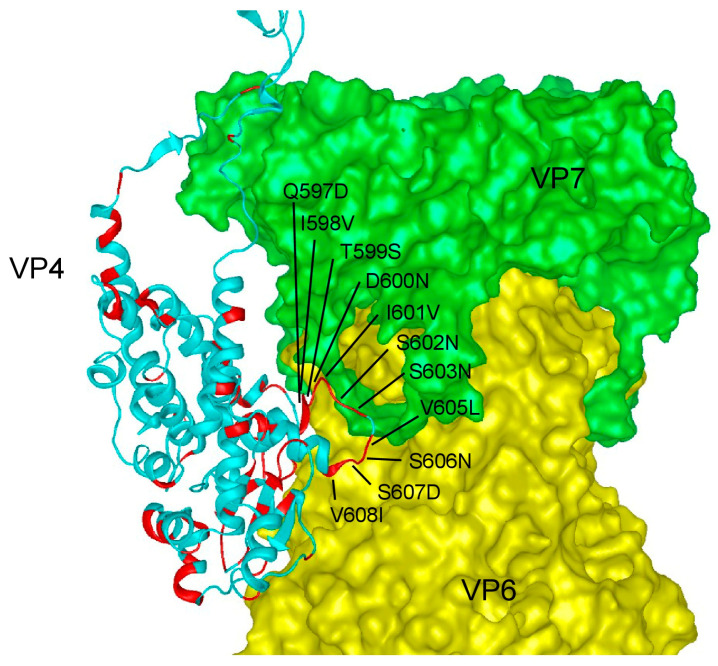
Location of the identified amino acid differences between VP4-SA11 and VP4-Wa in the VP4 foot. Amino acid differences were mapped onto the atomic model of an infectious rhesus rotavirus particle on the basis of PDB 4V7Q [5]. A side view of a VP4 monomer (chain BX) with a VP7 trimer (chains BO, BP, and BQ) and a VP6 trimer (chains AL, AM, and AN) is shown. Amino acid residues in VP4-Wa differing from VP4-SA11 are highlighted in red. Amino acid changes in a VP4 loop close to VP7 and VP6 are labeled.

VP4 contains a head region, a body and stalk region, and a foot region (Figure S5a). The VP4 foot is located at the base of the VP4 spike and interacts with VP7 and VP6, while the VP4 body and stalk region lifts the VP4 head away from the virus particle. VP4 from SA11 and Wa are only identical at 554/776 (71%) amino acid residues (Figure S5b). The majority of differences are in the VP4 head, which contains the putative receptor-binding site (Figure S5a,b). However, eleven amino acid differences (Q597D, I598V, T599S, D600N, I601V, S602N, S603N V605L, S606N, S607D, and V608I) map to a loop in the VP4 foot that is in proximity to VP6 and to the N-terminal VP7 arm that locks VP7 to VP6 (Figure 6).

## 3. Discussion

The recently established plasmid-based RG systems for rotaviruses have developed as powerful tools for investigating basic and applied research questions [36]. Especially for the development of novel vaccines, RG systems may serve as a platform for the rapid integration of newly occurring antigen variants into vaccine strains without the need for difficult cell culture isolation trials of circulating strains [28,29]. Although it has been shown that, in principle, several capsid protein variants can be successfully integrated into the well-developed SA11-based RG system, limitations have been reported, especially for several VP4 variants of human strains [20,27,28,29,30]. We have recently shown that the exchange of the receptor-binding region of VP4 from human RVAs with the corresponding region from SA11 can overcome this problem [31]. However, this exchange may affect immunogenicity of the protein, which would be a disadvantage for vaccine development. Here, we show that the co-exchange of VP7 and VP6 can restore infectivity of a human VP4 reassortant, which might pave the way for the efficient generation of SA11-based reassortants carrying unaltered antigens of heterologous human strains.

We were not able to rescue the Wa-VP4 mono-reassortant or the VP4/VP7-Wa and VP4/VP6-Wa double-reassortants in the backbone of SA11. The underlying reasons are not known so far. Strain-specific RNA–RNA interactions of the respective genome segments and/or of their expressed products might influence reassortment [16,37,38]. As our triple-reassortant in the backbone of SA11 replicated well, general incompatibilities of the RNA segments seem to be unlikely, although specific interactions between the exchanged genome segments cannot be completely ruled out. As several amino acid exchanges between Wa and SA11 at the protein interfaces of VP6, VP7, and VP4 have been identified, unfavorable protein–protein interactions might cause defects in mono- and double-reassortants. This is supported by results from Pesavento et al., who examined the VP4 spike structure in a series of simian and bovine RVA strains carrying combinations of heterologous VP4 and VP6 from bovine or simian RVA strains [39]. In certain reassortants, subtle but visible alterations in the VP4 conformation were observed by electron cryomicroscopy, which correlated with unusual VP4-associated phenotypic changes. 

Our analyses of the capsid structures by TEM showed no alterations in the capsids of the generated reassortants. Although the applied technique was not able to resolve the structures of VP4 in those capsids, it allowed the determination of rotavirus particle size. Non-infectious DLPs, which can form as an intermediate product during assembly [40], following uncoating [41] or when VP2 and VP6 are overexpressed in cells [42], are generally smaller than TLPs. Their presence could hint at incompatibilities between capsid proteins. Based on three-dimensional reconstructions from electron micrographs of frozen hydrated virus particles, SA11 TLPs were previously reported to be 76.5 nm in diameter (without the mostly invisible VP4 spike) [32], which corresponds well with our measurements. The overall presence of TLPs indicates that the recovered reassortants, including the triple-reassortant, generate virus particles comparable to the parent viruses. 

Kawagishi et al. and Sanchez-Tacuba et al. have shown that SA11 reassortants carrying VP4 from other tissue culture-adapted human RVA strains replicated dramatically worse than their respective parent human strains [20,27]. This could also indicate that the correct binding of VP4 within the VP7/VP6-pocket might be necessary for full function of this protein. This is supported by the data from the growth kinetics of our triple-reassortant containing both of these proteins, which showed comparable growth like the parent Wa strain again. It would be interesting to analyze the VP4 spike structure from poorly replicating or non-rescuable reassortants carrying VP4 from human RVAs, if sufficient amounts of those particles could be generated, to confirm that structural VP4 alterations correlate with the loss of infectivity. 

It has to be mentioned that the strain-specific interactions of VP4, VP7, and VP6 seem not to be the only factors restricting the generation of replicating reassortants. For example, Kanai et al. tested the generation of reassortants carrying combinations of VP4, VP7, and VP6 from human RVA strain U14 in the backbone of SA11 [28]. In their rescue experiments, a poorly replicating mono-reassortant carrying U14-VP4 was recovered, while rescue attempts of the VP4/VP7/VP6 triple-reassortant were unsuccessful. Therefore, restoring the natural VP4/VP7/VP6 interactions may not necessarily be sufficient to rescue combinations of other rotavirus strains. Kanai et al. suggested that incorrect interactions between VP6-U14 and VP2-SA11 might have been responsible for the inability to rescue their triple-reassortant. In contrast, VP6-Wa and VP2-SA11 seemed to be compatible in our study. An additional difference to our study is that attempts to culture the original U14 strain in MA-104 cells were unsuccessful, whereas we used the cell culture-adapted Wa strain.

In conclusion, we have shown that by restoring the interactions of VP4/VP7/VP6 from the human RVA strain Wa, we were able to generate reassortant SA11 with replication kinetics similar to the parent human RVA strain Wa. The results shed some light into RVA reassortment limitations and solutions to overcome them, but further research is required to determine the specific restrictions of capsid protein interactions at the amino acid level. In addition, it should be analyzed whether the finding is specific for the used combination of strains Wa and SA11. If it can be also applied to other RVA strains, the generation of triple-reassortants may represent one opportunity to develop novel well-replicating vaccine strains based on capsid protein genes cloned from human RV samples without affecting their antigenic structure.

## 4. Materials and Methods

### 4.1. Cell Lines and Viruses

The BSR-T7/5 cells were kindly provided by Karsten Tischer (Free University of Berlin, Germany) and MA-104 cells were retrieved from the European Collection of Authenticated Cell Cultures (Salisbury, UK). All cell culture reagents and cell culture conditions have been described previously in detail [29]. The virus strain RVA/Simian-tc/ZAF/SA11-L2/1958/G3P[2], referred to as SA11 throughout the manuscript, was generated using the plasmid-based RG systems as described below. The tissue culture-adapted virus strain RVA/Human-wt/USA/Wa/1974/G1P[8], referred to as Wa throughout the manuscript, was retrieved from the American Tissue Culture Collection (VR-2018, Manassas, VA, USA) through LGC Standards Ltd. (London, UK). The genotypes of the eleven genome segments from SA11 and Wa are shown in Table 1.

### 4.2. Plasmids

The plasmids encoding the eleven SA11 genome segments as well as the three helper plasmids pCAG-D1R, pCAG-D12L, and pCAG-FAST-p10 were a kind gift from Takeshi Kobayashi [25] and obtained from Addgene (Watertown, MA, USA). The plasmids carrying the Wa genome segments contained an expression cassette consisting of the T7 RNA polymerase promoter, the complete genome segment, a hepatitis delta virus ribozyme sequence, and a T7 RNA polymerase terminator. The sequences for the promoter, ribozyme, and terminator are identical to a plasmid encoding VP4 from avian RVA strain 02000V2G3 [43]. The generation of the plasmid encoding VP4 from the human RVA strain Wa has been described previously [30]. The VP7-Wa and VP6-Wa expression cassettes with suitable restriction sites at the 5′ and 3′ ends were synthesized as dsDNA fragments (gBlocks, Integrated DNA Technologies, Coralville, IA, USA). Adenosine overhangs were added using Takara Ex Taq (Takara Bio Inc, Kusatsu, Japan) and the fragments were cloned into pCR4-TOPO using a TOPO TA cloning kit (Thermo Fisher Scientific, Waltham, MA, USA) according to the manufacturer’s instructions. The correct sequence of the expression cassette was verified by Sanger sequencing (Eurofins Genomics GmbH, Ebersberg, Germany). The expression cassettes were then cloned into a previously generated plasmid encoding VP7 from the human RVA strain Moz308 [29] using compatible restriction sites. All plasmids were transformed into One Shot TOP10 Chemically Competent E. coli (Thermo Fisher Scientific, Waltham, MA, USA) and purified using the QIAfilter Plasmid Midi Kit (Qiagen GmbH, Hilden, Germany). 

### 4.3. Plasmid-Based RG System

The reassortant virus was essentially generated as described previously [35,36]. Briefly, 90% confluent BSR-T7/5 cells grown in 6-well plates were co-transfected with eleven plasmids encoding the individual rotavirus genome segments (2250 ng for the plasmids encoding NSP2 and NSP5; 750 ng for the remaining plasmids) and three helper plasmids encoding two vaccinia virus capping enzyme subunits (750 ng each), as well as a small membrane fusion protein (15 ng) using 30 µL TransIT-LT1 transfection reagent (Mirus Bio, Madison, WI, USA). The cells were incubated with the transfection mix for 24 h before they were washed twice with PBS, and fresh media without serum as well as trypsin (0.5 µg/mL final concentration, PAN-Biotech, Aidenbach, Germany) were added. Forty-eight hours later, MA-104 cells and trypsin (2.0 µg/mL final concentration) were added. After three days, the co-cultured cells including the cell culture supernatant were frozen at −20 °C and thawed once at room temperature (freeze/thaw supernatants). These freeze/thaw supernatants were clarified by low-speed centrifugation prior to passaging.

### 4.4. Passaging of Reassortant Virus

The reassortant virus was essentially passaged as described previously [35,36]. Briefly, trypsin (20 µg/mL final concentration) was added to clarified freeze/thaw supernatants from the RG system (2 mL per well). After 1 h at 37 °C, confluent MA-104 cells grown in 6-well plates were washed twice with PBS and the supernatants were added. The cells were incubated for 1 h at 37 °C before they were washed once with unsupplemented medium. Fresh medium (without serum) was supplemented with trypsin (2 µg/mL final concentration) and added to the cells. After 7 days of incubation, the cells were passaged again following the same protocol. 

### 4.5. qRT-PCR and RT-PCR

The viral RNA was extracted from clarified culture supernatants using the NUCLISENS easyMAG system (bioMérieux, Marcy-l’Étoile, France) and digested with RNase-free DNase (Roche, Basel, Switzerland) according to the manufacturer’s instructions. The viral RNA was detected by qRT-PCR using the primer pair RVA7-1F/Rota NVP3-R and the probe RVA7probe1, which were specific to RVA-NSP3, as described previously [44]. Genome copy equivalents (GCEs) were calculated by including a standard based on a dilution series of plasmid pT7-NSP3SA11. RT-PCRs were performed using the OneStep RT-PCR Kit (Qiagen) according to the manufacturer’s instructions. The primer sequences are listed in Table S1. RT-PCR products were analyzed on a 2% agarose gel. Prior to Sanger sequencing (Eurofins) or restriction digest analyses, amplicons were cleaned up using the Monarch DNA Gel Extraction Kit (New England Biolabs, Ipswich, MA, USA).

### 4.6. Replication Kinetics

Diluted supernatants containing 2 × 10^4^ GCEs or 1.5 × 10^3^ plaque-forming units (PFUs) were activated by incubation in culture media without serum in the presence of trypsin (20 µg/mL final concentration) at 37 °C for 1 h. Confluent MA-104 cells grown in 6-well plates were washed twice with 2 mL PBS and the activated viruses were added. The activated viruses and the cells were incubated at 37 °C for 1 h before the media with the remaining virus were removed. Afterwards, 3 mL of fresh media without serum containing trypsin (2 µg/mL final concentration) was added to the cells. Culture supernatant was collected at the indicated time points and analyzed by qRT-PCR as described above or plaque assays as described below. 

### 4.7. Plaque Assays

Serial virus dilutions were activated by incubation with trypsin (20 µg/mL final concentration) at 37 °C for 1 h. Confluent MA-104 cells grown in 6-well plates were infected as described above. After removal of the supernatant, the infected cells were overlaid with media containing 0.6% low-gelling-temperature agarose and 2 µg/mL trypsin. The infected cells were incubated for either 2 days (SA11), 6 days (476-Wa), or 7 days (Wa) before they were stained with neutral red (50 µg/mL, Carl Roth, Karlsruhe, Germany). 

### 4.8. Transmission Electron Microscope (TEM) Analyses

The cell culture supernatants containing viral particles were used for TEM analyses. The supernatants were adsorbed onto formvar/carbon-coated 400 mesh copper grids (Plano GmbH, Wetzlar, Germany) for 5 min. Excess liquid was absorbed with a tissue paper and the samples were fixed for 1 min with 2.5% glutaraldehyde (Science Services GmbH, Munich, Germany). The grids were then negatively stained using 2% uranyl acetate (Sigma-Aldrich, St. Louis, MO, USA) for 1 min. Excess liquid was removed with tissue paper and the grids were dried until imaging. Imaging was performed on Jeol 1400 TEM (Jeol GmbH, Freising, Germany) operated at 120 kV. Images were captured with a Veleta G2 camera (Olympus, Hamburg, Germany). Four separate areas of the grid were analyzed to assess sample homogeneity. ImageJ was used for size analysis [45].

### 4.9. Sequence Analyses and Protein Structure Visualization

Segment-specific genotypes were determined using the ViPR computing process [7,46,47,48]. Sequence alignments were performed using the Clustal Omega method as implemented in MegAlign Pro (DNASTAR Inc., Madison, WI, USA). Protein structures were visualized using Protean 3D (DNASTAR Inc.) on the basis of the published atomic model of an infectious rotavirus particle [5].

### 4.10. Statistics

The data are presented as mean ± standard deviation. To determine statistical significance, a two-tailed unpaired t test was used. Results with a *p*-value below 0.05, 0.01, or 0.001 were considered statistically significant and marked with one, two, or three asterisks, respectively.

## Figures and Tables

**Table 1:** Segment-specific genotypes of SA11 and Wa genome segments.

Strain	Genotype										
	VP7	VP4	VP6	VP1	VP2	VP3	NSP1	NSP2	NSP3	NSP4	NSP5
SA11	G3	P[2]	I2	R2	C5	M5	A5	N5	T5	E2	H5
Wa	G1	P[8]	I1	R1	C1	M1	A1	N1	T1	E1	H1

## Data Availability

The nucleotide sequences of the T7 RNA promoter, hepatitis delta ribozyme, and T7 RNA polymerase terminator used in this study are identical to the ones used in a plasmid containing the expressing cassette of VP4 from chicken RVA strain 02000V2G3, which is available online (GenBank: KT239165) [43]. The nucleotide sequences of the Wa genome segments (Genbank: KT694939–KT694949) [49] and SA11 genome segments (Genbank: LC333802–LC333812) are also available [50]. The atomic model of an infectious rhesus rotavirus particle is available online (PDB 4V7Q) [5].

## Supplementary Materials

The following supporting information can be downloaded at: https://www.mdpi.com/article/10.3390/ijms24065670/s1.
