# Supplementary material for: Strain-Specific Interactions between the Viral Capsid Proteins VP4, VP7 and VP6 Influence Rescue of Rotavirus Reassortants by Reverse Genetics
Source: Int J Mol Sci. 2023 Mar 16;24(6):5670. doi: 10.3390/ijms24065670 (PMC10054668; doi:10.3390/ijms24065670)
Supplement: Supplementary file 1 [file ijms-24-05670-s001.zip › ijms-2263729-supplementary.pdf]

Supplementary File

Strain-specific interactions between the viral capsid proteins VP4, VP7 and VP6 influence rescue of rotavirus reassortants by reverse genetics

Roman Valusenko-Mehrkens, Ashish K. Gadicherla, Reimar Johne, Alexander Falkenhagen*

Department of Biological Safety, German Federal Institute for Risk Assessment, Max-Dohrn-Str. 8-10, 10589 Berlin

*Corresponding author

Email contact:
Alexander.Falkenhagen@bfr.bund.de

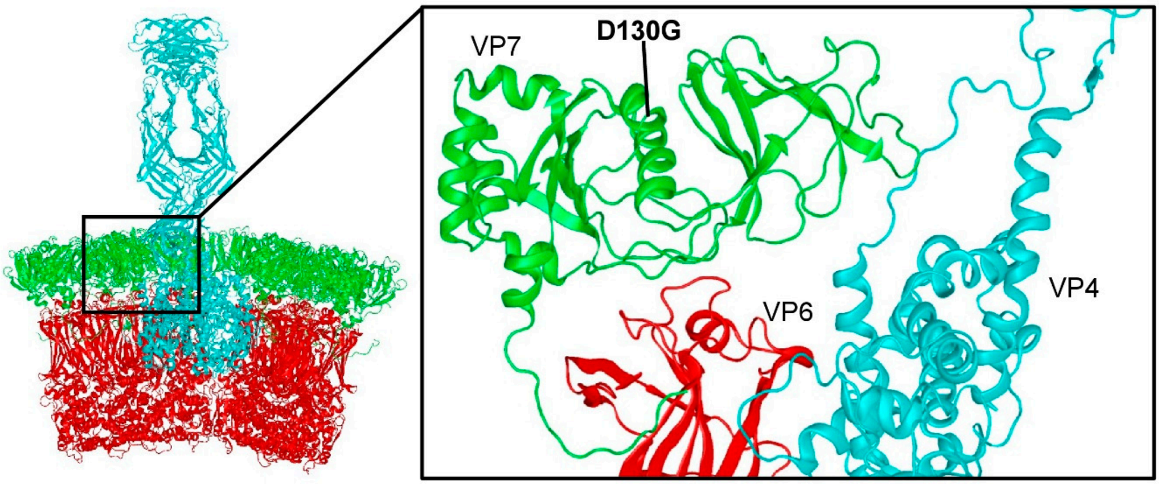

Figure S1. Position of the identified VP7 mutation in the atomic model of an infectious rhesus rotavirus particle (PDB 4V7Q, Settembre et al., 2011).

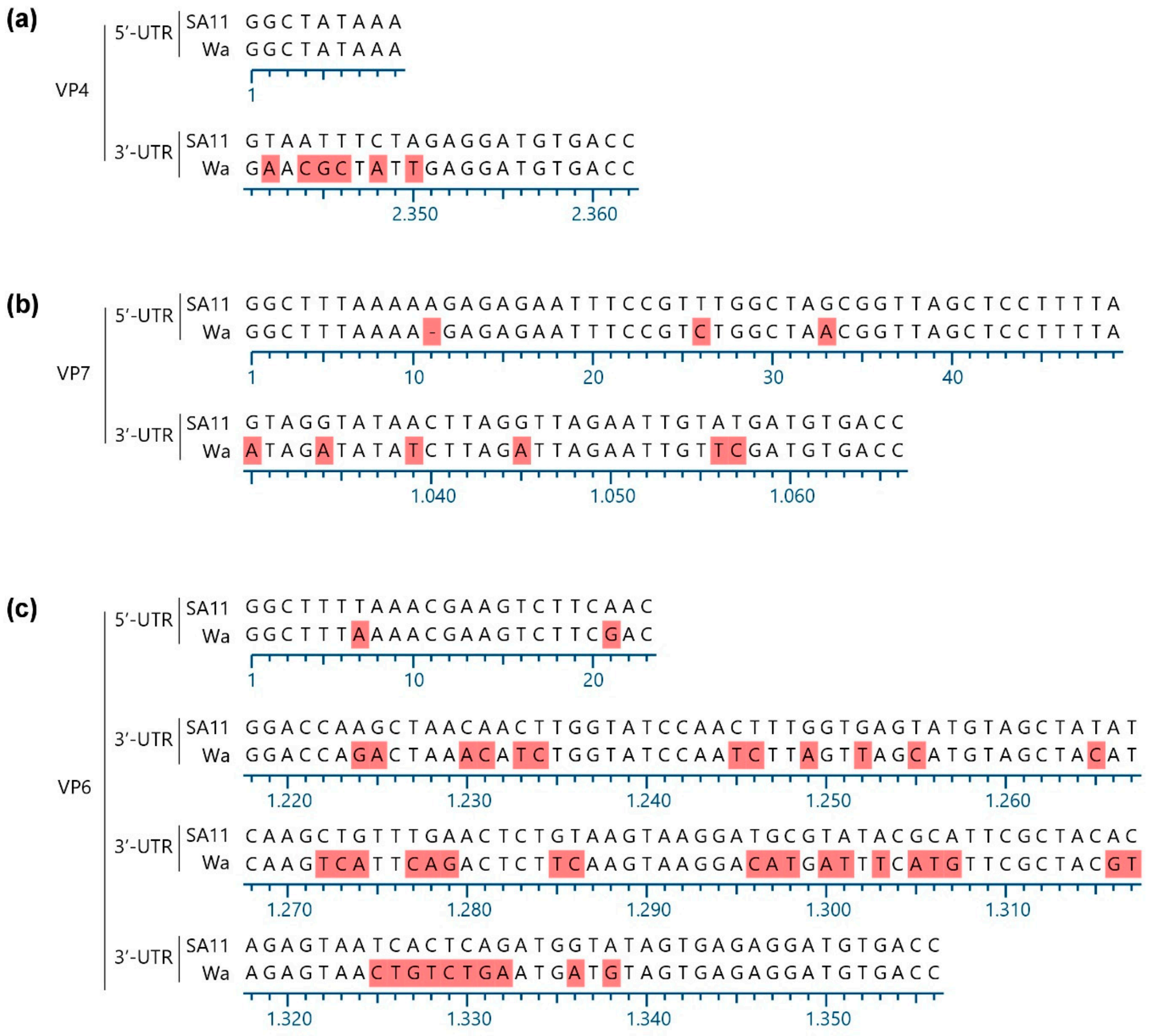

Figure S2. Alignment of the VP4, VP7, and VP6 UTRs**. (a)** VP4. **(b)** VP7. **(c)** VP6. Nucleotides in Wa differing from SA11 are highlighted in red.

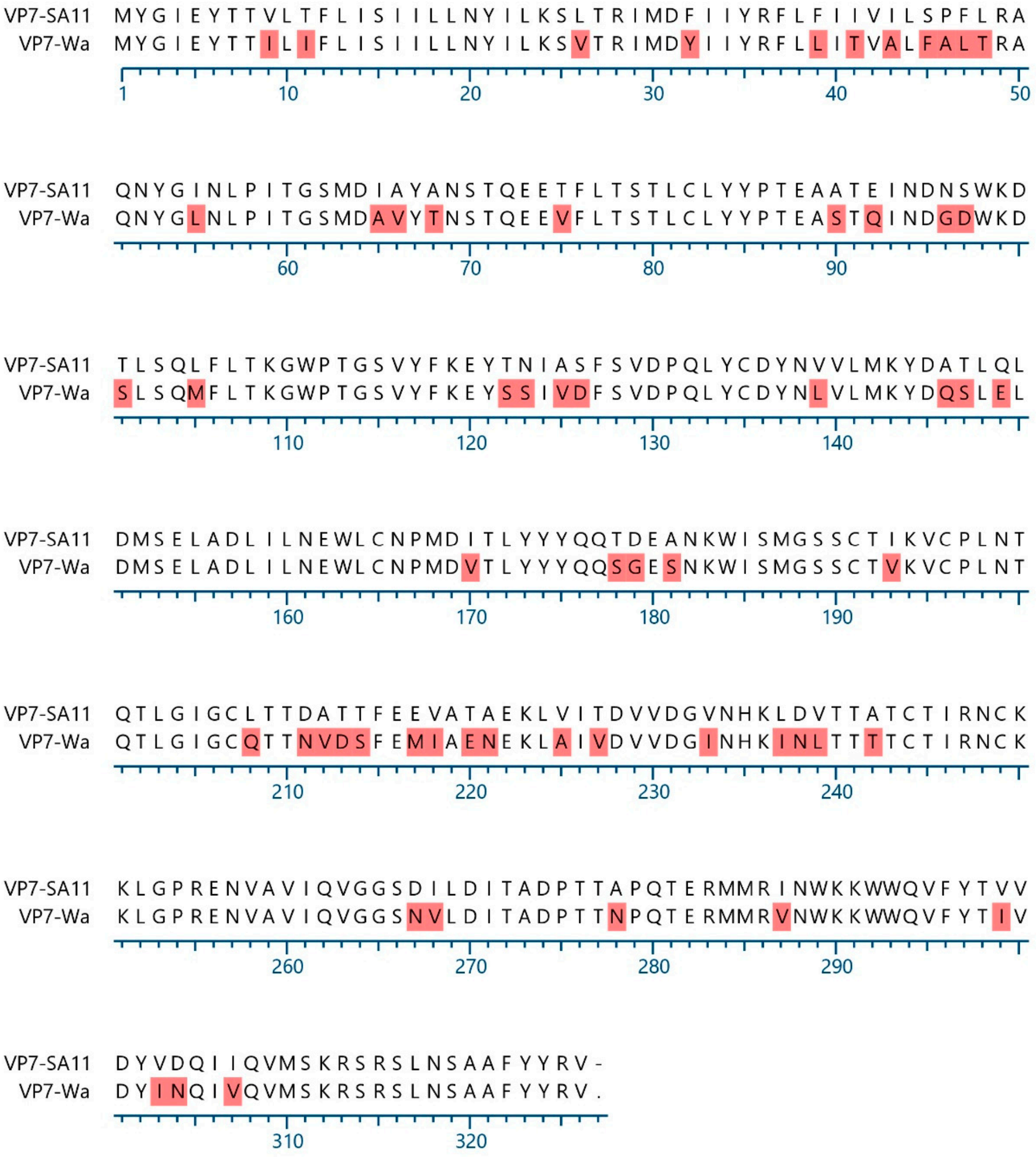

Figure S3. VP7 amino acid sequence alignment. Amino acid residues in Wa differing from SA11 are highlighted in red.

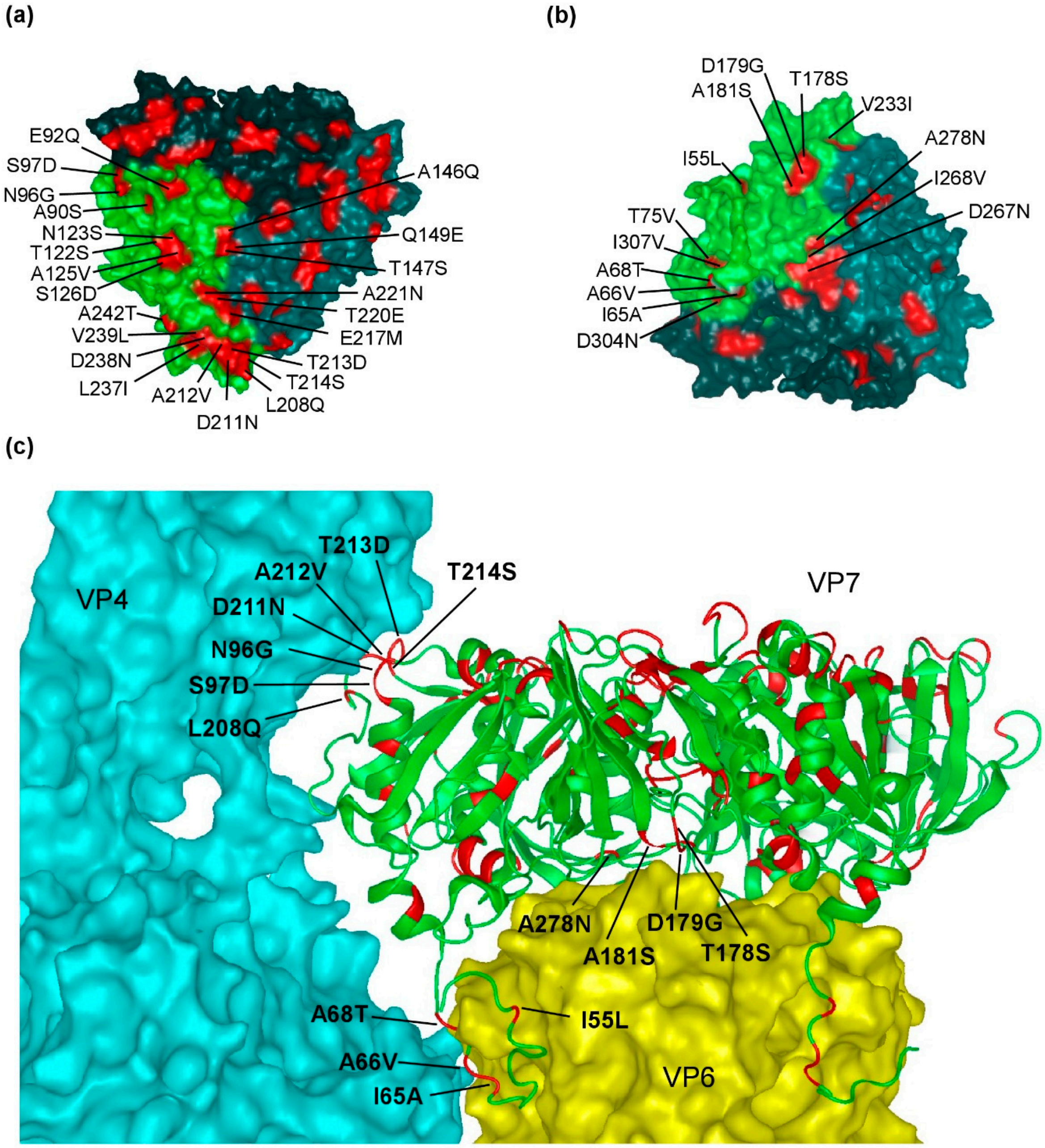

Figure S4. Location of the identified amino acid differences between SA11 and Wa in VP7. Amino acid sequence differences were mapped to the atomic model of VP7 (PDB 4V7Q, Settembre et al., 2011). Amino acid residues in VP7-Wa differing from VP7-SA11 are highlighted in red. **(a)** Outer surface of a VP7 trimer viewed from the top. VP7 monomers are colored in different shades of green. **(b)** Inner surface of a VP7 trimer viewed from the bottom. VP7 monomers are colored in different shades of green. **(c)** Side view of a VP7 trimer (chains BO, BP and BQ) in complex with the trimeric VP4 spike (chains BX, BY and BZ) and a VP6 trimer (chains AL, AM and AN). Amino acid changes in close proximity to VP4 or VP6 are labeled.

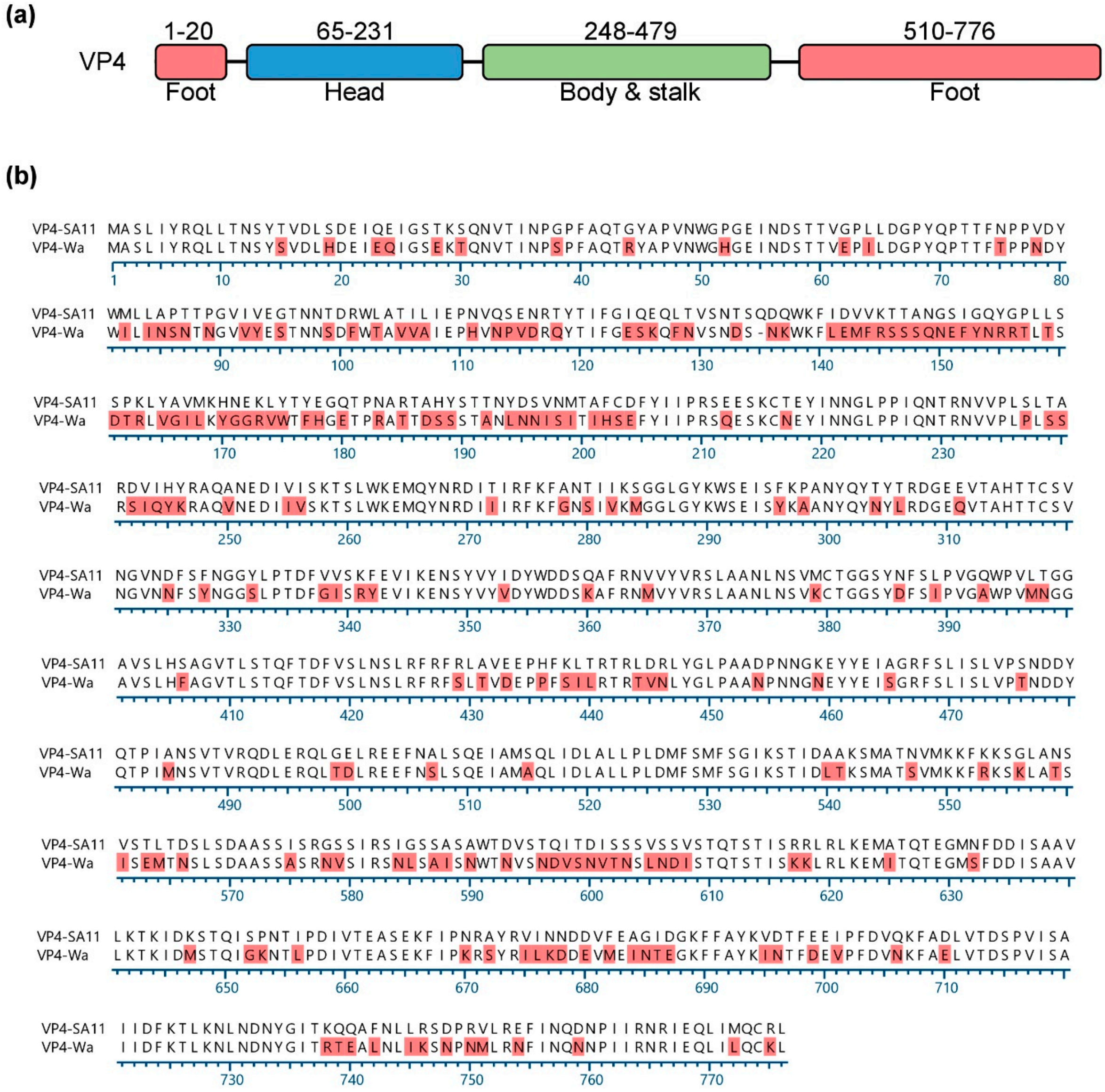

Figure S5. Amino acid differences between VP4-SA11 and VP4-Wa. **(a)** Diagram of VP4 showing the location of the head, body & stalk, and foot region. Amino acid residues contributing to each region are indicated (Settembre et al., 2011). **(b)** Amino acid sequence alignment of VP4-SA11 and VP4-Wa. Sequence differences are highlighted in red.

Table S1. Primers used in RT-PCR analyses

Primer name	Primer sequence
VP4-Wa-A1-F	5’-GCCAACTACATTTACTCCACCTAAT-3’
VP4-Wa-A1-R	5’-CTTCCACCATATTTCAATATTCCTACA-3’
VP7-Wa-A1-F	5’-ACTGTAGCATTATTTGCTTTGACA-3’
VP7-Wa-A1-R	5’-CGATATCCACTTATTTGATTCTCCC-3’
VP6-Wa-A1-F	5’-CGTTGAGAAAGTTAGCGGGA-3’
VP6-Wa-A1-R	5’-ATAGTAGCTGTAGTCAGTGCG-3’
VP2-SA11-F	5’-AATTTCCAATACCATCTATTACG-3’
VP2-SA11-R	5’-CTGGTATGTAATTGAAAATGATA-3’

References

Settembre EC, Chen JZ, Dormitzer PR, Grigorieff N, Harrison SC. Atomic model of an infectious rotavirus particle. EMBO J. 2011;30(2):408-416. doi:10.1038/emboj.2010.322
